# Determination of the Antioxidant Activity of Samples of Tea and Commercial Sources of Vitamin C, Using an Enzymatic Biosensor

**DOI:** 10.3390/antiox10020324

**Published:** 2021-02-22

**Authors:** Danilo Braga Ribeiro, Gabriela Santos Silva, Djanira Rubim dos Santos, Andressa Rose Castro Costa, Eliane Braga Ribeiro, Mihaela Badea, Gilvanda Silva Nunes

**Affiliations:** 1 Pesticide Residue Analysis Center, Federal University of Maranhão, UFMA. Av. Portugueses, CCET, Bacanga, CEP, São Luis, MA 65080-040, Brazil; dannilobraga15@hotmail.com (D.B.R.); gabriela.santos@discente.ufma.br (G.S.S.); djanira.rubim@discente.ufma.br (D.R.d.S.); andressa.rcc@discente.ufma.br (A.R.C.C.); eliane.ribeiro@discente.ufma.br (E.B.R.); 2 Center for Fundamental Research and Prevention Strategies in Medicine, Department of Fundamental, Prophylactic and Clinical Specialties, Transilvania University of Brasov, 500039 Brasov, Romania

**Keywords:** antioxidants, biosensors, xanthine oxidase, teas, drugs, vitamin C

## Abstract

Antioxidants are synthetic or natural compounds capable of preventing or delaying oxidative damage caused by chemical species that can oxidize cell biomolecules, such as proteins, membranes, and DNA, leading to the development of various pathologies, such as cancer, atherosclerosis, Parkinson, Alzheimer, and other diseases serious. In this study, an amperometric biosensor was used to determine the antioxidant activity of teas and effervescent products based on vitamin C, available on the market. A sensor composed of three electrodes was used. The performance of the following electrochemical mediators was evaluated: meldola blue combined with Reineck salt (MBRS), Prussian blue (PB), and cobalt phthalocyanine (CoPC), as well as the time of polymerization in the enzymatic immobilization process and the agitation process during chronoamperometric measurements. Prussian blue proved to be more efficient as a mediator for the desired purposes. After optimizing the construction stages of the biosensor, as well as the operational parameters, it presented stability for a period of 7 months. The results clearly indicate that the biosensor can be successfully used to detect fraud in products called “antioxidants” or even in drugs containing less ascorbic acid than indicated on the labels. The detection limit was set at 4.93 µmol·L^−1^.

## 1. Introduction

Due to redox reactions that provide cells with the energy necessary for their functioning, external factors such as pollution, bad habits (smoking, alcohol consumption), and inadequate nutrition cause an increase in formation of free radicals in the human body [1]. 

Diabetes, cirrhosis, cardiovascular diseases, some types of cancer, and neurological disorders are examples of diseases often associated with irregular and uncontrolled processes in the production of these radicals. In excess, they can cause oxidative damage to cells, forming advanced glycation products, inactivating proteins (enzymes), and attacking membrane lipids, carbohydrates, and DNA [2,3,4].

The search for new methods of evaluating the antioxidant activity of several compounds potentially capable of inhibiting such damage in biological systems or even in food has increased in the last years, as shown by a search in the Web of Science database on 4 February 2021 (Supplementary Data)—1917 references since 1997 for antioxidant capacity detection, most of them from the last five years. From all the indicated references, 1801 (93.95%) were articles, 86 (4.46%) were proceedings papers, and 81 (4.23%) reviews. Considering the journals that published these papers, most of them are dealing with applications in food chemistry. Considering the entire group of proposed articles, an H-index—112 was generated, indicating in this way the importance of this topic for scientific media. We believe that also our study will be an important one due to the applications proposed in the sample of tea and commercial sources of vitamin C, using a novel methodology developed recently by our group and previously successfully applied for testing antioxidant capacity of fresh and frozen fruit. Of natural or synthetic origin, antioxidants are able to prevent or delay oxidative damage generated by oxidizing sources, even when in lower concentration compared to the oxidizable substrate [5]. The human body’s antioxidant defense system consists of a range of bioactive compounds capable of neutralizing the action of free radicals, such as vitamins (A, C, E, K), glutathione, mineral salts, metalloproteins, enzymes (SOD—superoxide dismutase) and polyphenols [6,7]. Antioxidants of exogenous origin come from plant sources, such as fruits and teas, and are also found in commercially available supplements [8,9,10]. 

Over the years, the use of plants for medicinal purposes has grown considerably, either empirically or in complex compositions of the pharmaceutical industry and consumption in the form of teas has been quite expressive due mainly to the incentive to use natural products.

Different methodologies based on electrochemical, spectrophotometric, and chromatographic techniques have been described in the literature in order to assess the antioxidant capacity in different matrices. In general, they differ in terms of the mechanisms for obtaining oxidizing species and in the way the final products are measured. However, they have the drawback of being relatively time consuming and employing expensive bio-reagents [11,12,13]. In addition, due to the presence of unsaturation in its chemical structure, antioxidants from natural products can present stability problems that make them sensitive to exposure to heat, light, and the presence of oxygen [14].

On the other hand, in recent years, electrochemical biosensors have been considered as a promising tool in determining the antioxidant potential, due to their characteristics as selectivity, low cost of obtaining, ease of storage, miniaturization capacity, easy automation, and portability, which combined make possible in situ analysis, reducing the risk of interference resulting from the destabilization of compounds [15,16].

Therefore, this work aims to improve and apply an analytical device in the determination of the antioxidant capacity in samples of teas and commercial sources of vitamin C, which offers the additional advantages of facilitated construction, high precision, and sensitivity of detection and that allows use both in the laboratory and in situ. In this paper, electrochemical evaluations will be performed using the cyclic voltammetry (CV) technique and chronoamperometric measurements, comparing with the previous paper [12] by our group where amperometry was used.

## 2. Materials and Methods

### 2.1. Reagents and Solutions

All reagents used were of analytical grade and the water used was deionized (Milli-Q Millipore 18.2 MΩ cm^−1^). Prussian blue or ferric ferrocyanide (PB) was obtained from Gwent Group (Torfaen, United Kingdom). Water-soluble polyvinyl alcohol photopolymer (PVA-AWP) was purchased from Toyo Kogyo Corporation (Chiba, Japan). Monobasic potassium phosphate (KH_2_PO_4_), dibasic potassium phosphate (K_2_HPO_4_), potassium chloride (KCl), hypoxanthine (HX), bovine milk xanthine oxidase enzyme (XOD) were all purchased from Sigma Aldrich Corporation (Nasdaq-Sial, Darmstarm, Darmst, Germany). Ascorbic acid (C_6_H_8_O_6_) was purchased from Merck (Seelze, Germany). The 50 mmol·L^−1^ K-PBS buffer solution (K_2_HPO_4_ 33.33 mmol·L^−1^, KH_2_PO_4_ 16.67 mmol·L^−1^) containing 10 mmol·L^−1^ KCl (pH 7.5) was used in the preparation of the enzymatic solutions (stock and work), solutions of the substrate hypoxanthine (HX) 5 mmol·L^−1^ and as the electrolyte in electrochemical measurements. It was also used as a solvent in tea infusions and dissolution of effervescent vitamins C.

### 2.2. Instrumentation

The electrochemical measurements using the biosensors were made in a a Ivium-n-stat potentiostat/galvanostat controlled by the IviumSoft software (Ivium Technologies, Eindhoven, Netherland). The working, reference, and auxiliary electrodes were printed on a thin transparent polyvinyl chloride (PVC) plate, which constituted the electrochemical sensor. The reference pseudo electrode was constituted by a straight line 5 × 1.5 mm in diameter, and is formed by a mixture (paste) of Ag/AgCl. The working electrode consisted of a 4 mm diameter disk, formed by a commercial graphite paste containing Prussian blue salt (PB) or Meldola blue with Reinecke salt (MBRS) or cobalt phthalocyanine (CoPC) as a modifier. The auxiliary electrode, formed by a 16 × 1.5 mm curved line, contained only the commercial graphite paste. Screen-printed electrodes (SPE) were produced in laboratory of University of Perpignan via Domitia, using a DEK 248 printing machine, and offered for these studies by Prof. Dr. Jean-Louis Marty.

### 2.3. Electrochemical Characterization

Electrochemical evaluations of sensors and biosensors were performed using the cyclic voltammetry (CV) technique. The biosensors were initially prepared according to the methodology developed in our research group [12]. An enzymatic charge of 8 mU XOD was immobilized under the surface of the modified working electrode, from the deposition of 3 µL of a homogeneous mixture of the enzymatic solution and PVA- AWP in the proportion 1:2 and later polymerization under neon light at 4 °C for 30 min. The effect of pH on the biosensor response was also evaluated.

### 2.4. Chronoamperometric Measurements and Parameter Optimization

All chronoamperometric measurements were performed at room temperature using a 10 mL dark electrochemical cell. The biosensor was previously subjected to 10 voltametric cycles and the current generated was then measured at a fixed working potential of −100 mV vs Ag/AgCl, where there is a reduction in H_2_O_2_.The intensity of the initial current was recorded after swelling of the PVA-AWP, followed by signal stabilization, in a total time of approximately 20 min. Then, an analytical curve was constructed by successively adding aliquots of the HX 5 mmol·L^−1^ solution under constant stirring. Then, the polymerization time (30 and 60 min) and the stirring conditions during measurements were optimized, as well as the current measurement time.

### 2.5. Determination of the Antioxidant Capacity of Real Samples

As a negative control, the production of reactive oxygen species (ROS) was used without neutralizing them by antioxidants. An analytical curve of current intensity (at the end of 75 s) as a function of the concentration of the hypoxanthine substrate (HX) was constructed, and the angular coefficient recorded (ma). Then, a new analytical curve was built, but in the presence of the antioxidant solution (standards or samples), and the antioxidant potential of the solution or sample was determined. The antioxidant capacity was expressed by the percentage of ROS inhibition by comparing the slope obtained in the curves constructed in the absence (ma) and the presence of antioxidants (mb), according to the Equation (1):

(1) $$Antioxidant\;capacity\;\%=100*\left[1-\left(\frac{mb}{ma}\right)\right].$$

#### Sample Preparation and Analysis

Samples of teas (fennel, chamomile, mint, cimegripe tea) and effervescent vitamin C were obtained from pharmacies in the city of São Luís, Maranhão, Brazil.

In assessing the antioxidant capacity of the tea, a volume of 50 mL of an infusion in K-PBS buffer was prepared by heating for 10 min on a magnetic stirrer with heating. A 10 mL volume of the infusion was transferred to the electrochemical cell. Then, analytical curves were constructed in the presence of HX in different concentrations, and the slopes of the curves constructed in the presence and absence of the samples were compared.

The samples of effervescent vitamin C were prepared by dissolving the mass of the tablet corresponding to 500 mg of ascorbic acid in 20 mL of K-PBS buffer. An aliquot of 20 µL of this solution was then transferred to the electrochemical cell, and the volume was made up to 10 mL with K-PBS. The curves were constructed, according to the procedure described above, and the antioxidant capacity determined.

The experiments were carried out using an infrared spectrometer from Shimadzu, model IR-Prestige-21 with an extended KBr (potassium bromide) beam splitter. The data were collected in a range of 500 to 4000 cm^−1^, at a resolution of 4 cm^−1^ using a spectral medium of 40 scans in potassium bromide.

The content of ascobic acid in the drugs was determined by the standard addition method. Then, 100 µl aliquots of the samples were transferred to a 50 mL volumetric flasks and volumes (500, 1000, 1500, 2000, and 2500 µL) of a 1 mg·L^−1^ solution of pure ascorbic acid was added to them, completing them with water. The absorbance was measured at a wavelength of 264 nm, using a UV-VIS spectrophotometer (Themoscientific, Orion AquaMate 8000), quartz cuvette 1 cm of optical path.

## 3. Results and Discussions

### 3.1. Electrochemical Characterization

The sensors used were built on a flexible and chemically inert base (PVC), where the three electrodes were printed, using a simple methodology based on semi-automatic screen printing. Such technology enables the manufacture of economic, portable, quick-response electrodes, with high sensitivity, low power required, disposable, and with the ability to operate at room temperature, thus enabling the performance of in situ analyses [17].

The O_2_ and H_2_O_2_ molecules monitored in the system proposed here are electroactive species that undergo oxidation and/or reduction when subjected to high work potentials, generating an electrical signal. Uric acid, the product of the enzymatic reaction, as well as several antioxidant compounds (ascorbic acid, for example), are also oxidized when they occur at high potentials, which can generate interference in the measured current [18]. Thus, the use of electrochemical mediators aims to annul or reduce such interference since it allows working with lower potentials [19]. Mediators are chemical species capable of donating or receiving electrons, thus helping to regenerate the oxidation state of the enzyme and its active center in an enzymatic reaction. The modifying agent has the function of increasing the sensitivity of the electrodes and can be incorporated into the carbon paste by directly adding a certain mass of the modifier in a mixture of graphite powder and binder [20]. 

**Figure 1:**
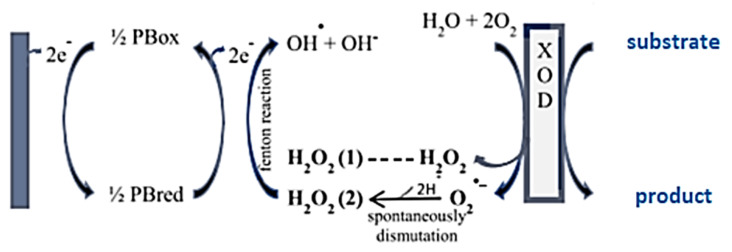
Principle of detection using XOD based biosensor, using Prussian Blue (PB), as mediator.

The generated H_2_O_2_ is reduced on the polarized (−100 mV vs. Ag/AgCl) WE surface, in presence of PB mediator, which has a specific catalytic effect for the H_2_O_2_ reduction due to its structure [21]. The O_2_•- radicals and/or H_2_O_2_ are scavenged with a decrease of the cathodic current which permits the quantification of the antioxidant capacity of different samples. In the Figure 1, the principle of detection using XOD based biosensor, using PB as mediator, is shown.

It is important to note that a range of working potential between 0 and −200 mV is quite desirable, when the focus of the electrochemical system is the determination of antioxidant capacity since at potentials below −200 mV there is a reduction in molecular oxygen, that at potentials above 0 mV oxidation of antioxidant compounds occurs [12,22].

**Figure 2:**
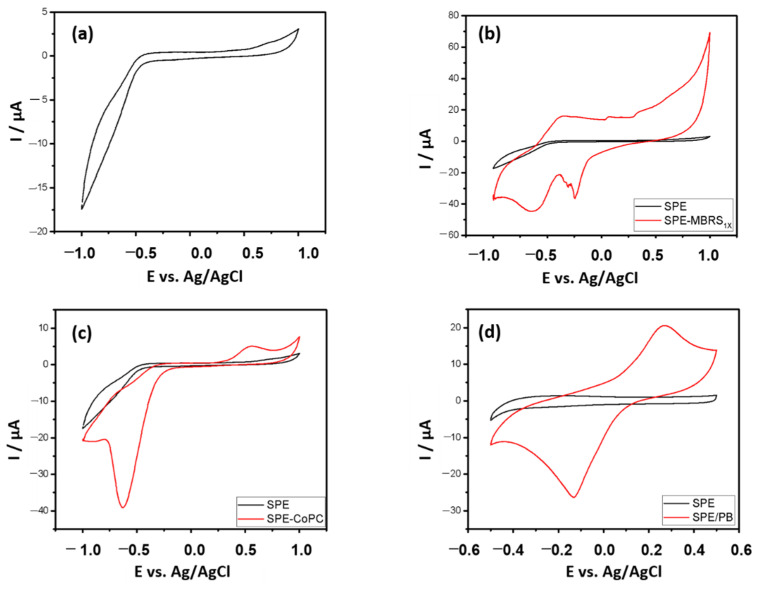

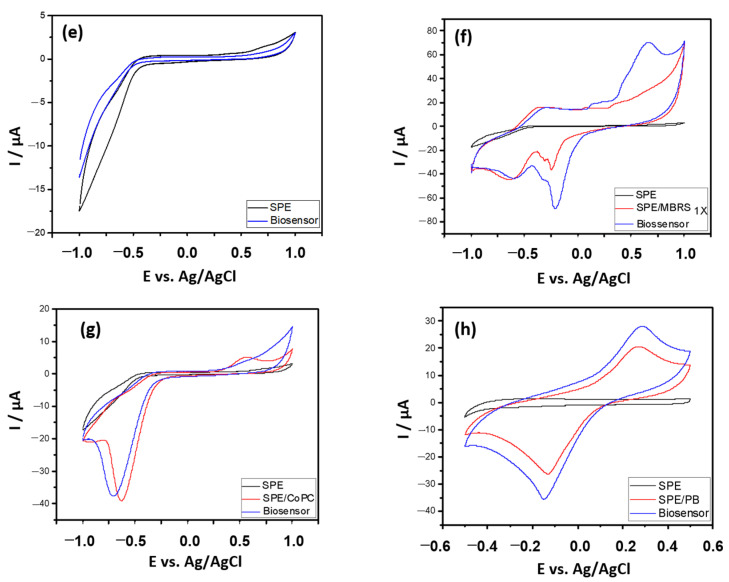
Cyclic voltamogram of (**a**) carbon paste electrode (SPE), (**b**) SPE modified with meldola blue with Reinecke salt (MBRS), (**c**) SPE modified with Cobalt phthalocyanine (CoPC), (**d**) SPE modified with PB and biosensors (**e**) without mediator and mediated with (**f**) MBRS, (**g**) CoPC, and (**h**) (PB) in K-PBS 50 mmol·L ^−1^ pH 7.5. Scan rate 50 mV·s^−1^.

Figure 2 shows the cyclic voltammetry of printed electrodes of carbon paste, bare and modified, as well as the electrochemical response after immobilization of the enzyme xanthine oxidase. 

There is a limitation for the printed carbon paste electrode in the cathodic region at potentials below −0.5 V and anodic above 0.5 V vs. Ag/AgCl, regions in which the supporting electrolyte is discharged. However, such behavior did not produce interference, as it is outside the desired potential window.

The use of Prussian blue (PB) as a mediator proved to be feasible for application in determining the antioxidant capacity, as it has a cathodic peak at −133 mV, within the desired potential window (0 to −200 mV). An increase in cathodic and anodic current was also observed with the immobilization of the enzyme, indicating that the electronic transfer process was favored, demonstrating an electrochemical affinity between the enzyme and the mediator. In addition, the incorporation of the PB mediator into the working electrode has been described as a simple, cost-effective, and highly stable process in acid and neutral media.

The intensity of cathodic and anodic current obtained with the biosensors when MBRS was the mediator was greater, demonstrating that there is an electrochemical affinity between the enzyme and this mediator as well. In the case of CoPC, in addition to not having seen such an increase, there was also a negative shift in the cathodic peak potential. It is also noted that, in the region of interest, there is no electrocatalytic activity, making its use unfeasible in this study.

**Figure 3:**
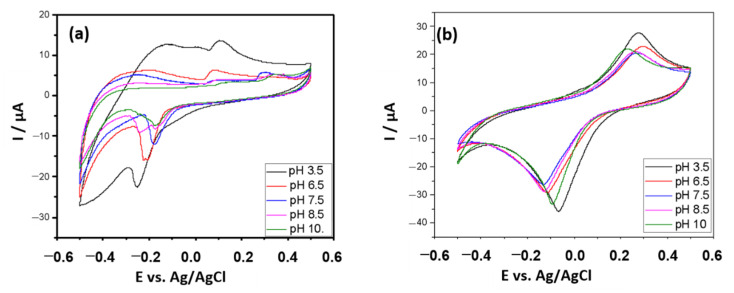
Evaluation of the effect of pH on the electrochemical behavior of the biosensor modified with (**a**) MBRS and (**b**) PB in K-PBS buffer as a function of the mediator’s immediate behavior. Scan rate: 50 mV·s^−1^.

In Figure 3, it can be seen that the electrochemical activity of PB is even more favored in an acidic environment. Due to the affinity of the enzyme with MBRS, its behavior at other pH was investigated, envisioning a possible application. However, considering the preservation of the enzyme activity, the medium with a pH close to neutrality was chosen as the working medium.

Oxidation processes can be favored or compromised at specific pH levels, generating different oxidation and reduction potentials (greater or lesser), as well as greater sensitivity (higher peak currents). For the MBRS mediator, despite its high efficiency in the electronic transfer process, proven in the present study, the negative shift in the cathodic peak potential at pH 3.5 and pH 6.5 disadvantaged its use. In a more alkaline environment, there is a decrease in the cathodic current and the formation of ill-defined peaks, which may be possibly caused by problems in electronic kinetics.

It is also noteworthy that the catalytic properties of Prussian blue on the reduction of hydrogen peroxide are well known and have been discussed previously by several researchers [23,24,25].

### 3.2. Biochemical Principles and Electrochemical Characterization of the Biosensor

The detection principle explored in the present work was based on the measurement of the H_2_O_2_ reduction current generated as a final product of the hypoxanthine (HX) oxidation reaction to uric acid, catalyzed by the XOD enzyme. The current was proportional to its concentration. Antioxidants inhibit such ROS, causing a decrease in the cathodic current, thus evaluating the antioxidant capacity.

**Figure 4:**
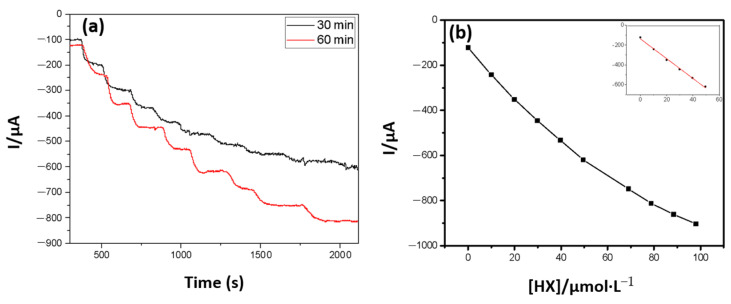
(**a**) Response of the amperometric biosensor with the successive addition of HX under constant agitation of 300 rpm, with the PB as a mediator and 8 mU of immobilized enzyme load, with polymerization times of 30 and 60 min. E = −100 mV. (**b**) Calibration curve of the amperometric biosensor modified with PB, when polymerization time of 60 min is used.

In accordance with the methodology described in the experimental part, analytical curves were constructed (Figure 4), evaluating the signal obtained when using different polymerization times in the enzymatic immobilization step, and a calibration curve was subsequently constructed. 

The reduction of hydrogen peroxide was more favored when a 60 min enzyme polymerization time was used in the graphite network, under neon radiation, becoming evident that the degree of polymerization depends on the time of exposure to neon light and interferes with enzyme retention in polymer and permeability of substrate and enzyme reaction products.

**Figure 5:**
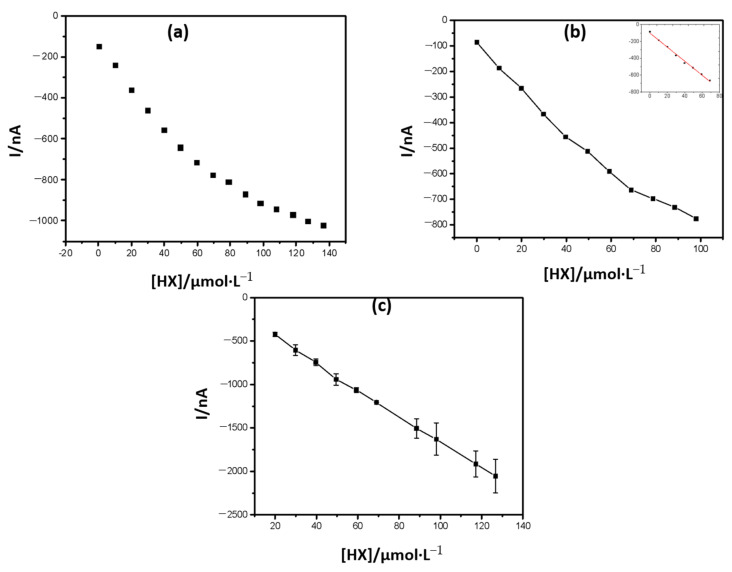
Curves obtained under different conditions: (**a**) 15 s of agitation, 15 s of rest, and 75 s measuring the current, fixed the time of 60 min of polymerization, (**b**) 30 s of agitation, 15 s of rest, and 60 s measuring the current, fixed the time of 60 min of polymerization, and (**c**) 15 s agitation, 15 s rest, 60 s current measurement, fixed the time of 60 min of polymerization.

The agitation conditions also interfered with the biosensor response (Figure 5); therefore, the best conditions for carrying out the measurements were determined.

From an analytical point of view, a greater linear range of work allows numerous alternatives for using the prototype, due to the variability of antioxidant capacities Table 1. Linear range and similar sensitivities, in amperometric biosensors, using printed electrodes modified with PB were achieved, fixing the work potential at −100 mV [25,26].

The operating conditions were fixed at 15 s of agitation, 15 s of rest, and 60 s of current measurement and detection and quantification limits were set at 4.93 µmol·L^−1^ and 16.43 µmol·L^−1^, respectively. They were calculated by the ratio between the average of 10 blanks measurements and an inclination of the analytical curve, multiplied by a factor of 3 and 10, respectively [12].

### 3.3. Determination of the Antioxidant Capacity of Commercial Samples

#### 3.3.1. Tea Samples

**Figure 6:**
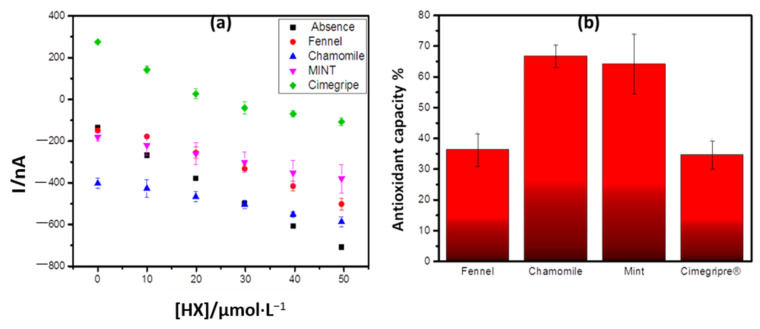
(**a**) Response of the biosensor in the presence of the antioxidant samples. (**b**)Antioxidant capacity.

As shown in Figure 6a, the initial currents recorded for the samples of chamomile tea and cimegripre^®^ tea, were discrepant in relation to that obtained in the absence of antioxidant and other samples. This may be the result of the different resistivity of the medium depending on the composition of the samples. However, it is noticeable in all cases the decrease in the cathodic current resulting from the reduction of H_2_O_2_ when compared to that obtained in the absence of the samples, resulting in smaller angular coefficients. Based on this difference, the total antioxidant capacity of the teas was calculated and the result shown in Figure 5b.

The wide variety of antioxidants, which may respond differently to the different oxidizing sources, makes it difficult to develop a single, simple, and universal method to assess antioxidant capacity, which is why different methods are generally used to characterize a sample [27].

The antioxidant capacity in the analyzed ones followed the order: chamomile > mint > fennel > cimegripe. This result is in agreement with that obtained by Nakamura et al. [28] when investigating the total antioxidant capacity of the infusions of chamomile, mint, and fennel teas by the CUPRAC (Cupric Reducing Antioxidant Capacity) method, based on the reduction of Cu^2+^ to Cu^1+^ when certain reducing agents are present in the medium, forming a complex of Cu^1+^/NC of intense color with maximum absorption at 454 nm, using the chromogenic reagent. This corroborates the applicability of the biosensor.

The importance of the study could be related also to the connection of these plants with their neuroprotective properties. In many countries, traditional herbal medicines are used to prevent or treat neurodegenerative disorders, and some have been developed as nutraceuticals or functional foods [29,30].

Fennel (*Foeniculum vulgare Mill*.) is a herbal that has antioxidant properties, with effects for prevention and treatment of stress-induced neurological disorders [31]. Fennel oil and trans-anethole, the main component of fennel oil, significantly inhibit SOCE-induced [Ca^2+^] increase in vascular endothelial cells and that these reactions may be mediated by NSC, IP3-dependent Ca^2+^ mobilization, and PLC activation [32]. Several clinical studies indicated fennel for its therapeutic potential to minimize neuronal toxicity by normalizing the expression levels of APP isoforms and oxidative stress markers [33]. Efficacy of oral fennel oil in the management of dysmenorrhea, premenstrual syndrome, amenorrhea, menopause, lactation, and polycystic ovary syndrome were confirmed according to results of clinical studies [34].

*Matricaria chamomilla* L. (chamomile) extract may produce clinically meaningful antidepressant effects in addition to its anxiolytic activity in subjects with a generalized anxiety disorder (GAD) and comorbid depression [35]. Chamomile had moderate antioxidant and antimicrobial activities, and significant antiplatelet activity in vitro. Animal model studies indicate potent anti-inflammatory action, some antimutagenic and cholesterol-lowering activities, as well as anti-spasmotic and anxiolytic effects [36].

Mints are aromatic plants traditionally used as a remedy and as culinary herbs. Methanolic extracts of Mentha x piperita and Mentha aquatica produced significant (*p* < 0.05) protection of the PC12 cells against oxidative stress. There were observed antioxidant and MAO-A inhibitory properties, M. x piperita being the most active. M. aquatica showed the highest affinity to the GABA(A)-receptor assay [37]. Its beneficial effect on the central nervous system as a neuroprotective potential, for example, has been explored. In addition, it targets multiple Alzheimer’s disease events [38].

Cimegripe^®^ is a mixture of paracetamol, clorfeniramina, and fenilefrina, with oral adult usage, for the relief of nasal congestion, runny nose, fever, and body pain present in flu-like states [39]. In the form of tea, its main ingredient is paracetamol. This medicine induces drowsiness, so it should not be used by vehicle drivers, machine operators, or those whose attention depends on the safety of others.

#### 3.3.2. Commercial Sources of Vitamin C

The enzymatic biosensor was used to determine the antioxidant capacity, not only of ascorbic acid (pure standard), but also of commercial effervescent vitamin C formulations.

Vitamin C provides protection against uncontrolled oxidation in the aqueous medium of the cell, due to its high reducing power, being a water-soluble and thermolabile vitamin. Humans and other primates, as well as the guinea pig, are the only mammals unable to synthesize it, requiring its administration through feeding or artificial supplementation [40].

**Figure 7:**
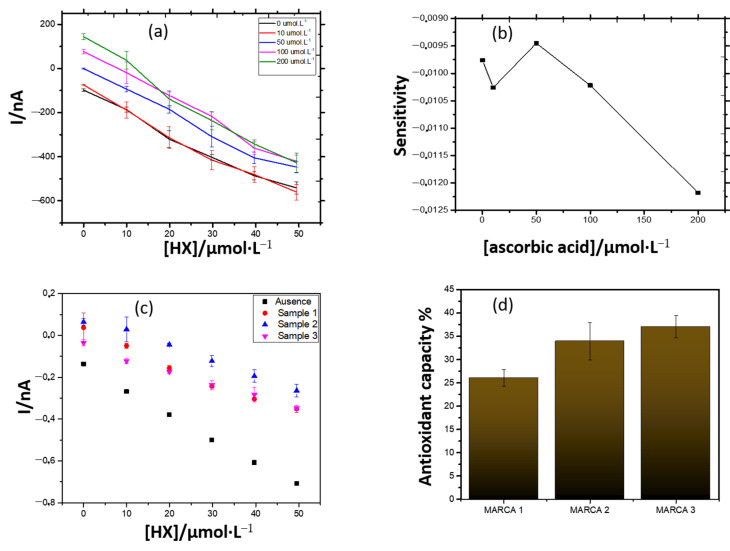
(**a**) Evaluation of the antioxidant capacity of ascorbic acid (standard) using the amperometric biosensor. (**b**) Sensitivity curve, in terms of the slopes of the curves showed in (**a**). (**c**) Response of the biosensor in the presence of the antioxidant samples. (**d**) Antioxidant capacity of drugs containing ascorbic acid against the biosensor.

Figure 7a shows the analytical curves constructed in the presence of different concentrations of ascorbic acid, in increasing concentrations of the HX substrate. By fixing the ascorbic acid concentration at 50 μmol·L^−1^, greater sensitivity was obtained and, in higher concentrations, a considerable loss of it (Figure 7b). A similar behavior was observed when samples of effervescent vitamin C were used as antioxidants in the system. For comparative purposes, the concentration of 0.53 mmol·L^−1^ of vitamin C was fixed, according to manufacturers’ specifications, and the antioxidant capacity of the effervescent vitamin C samples was determined (Figure 7c,d).

According to the manufacturers, each tablet of the effervescent contained 1 g of pure ascorbic acid. Thus, by weighing the equivalent quantity of each product, in order to make a final concentration of ascorbic acid equal to the three brands, it was expected that the results found for the antioxidant capacity were the same or very close. However, these were quite different from each other. The actual vitamin C content in the formulations may be the cause of the discrepancies found.

The composition of the samples was investigated by infrared spectroscopy and their spectra were similar, demonstrating that there are no significant differences between them. The content of ascorbic acid in the samples was determined by the standard addition method. Although samples 1 and 3 exhibited different antioxidant capacities, both contained the same content of ascorbic acid. This shows that the antioxidant capacity of the drugs is influenced by other compounds present in them. Some studies have shown a negative correlation between vitamin C content and antioxidant capacity [41,42]. Therefore, the individual and combined contribution of each component of the sample can be studied in the future.

The results of this study are in accordance with other published data using differential pulse voltammetry for vitamin C detection in pharmaceutical samples [43].

### 3.4. Analytical Stability of the Amperometric Biosensor

Adequate quality control is necessary to obtain reliable results. The use of control charts can be an effective strategy to ensure that there has been no change in a particular process over time. It helps to detect variations outside a statistically acceptable standard, making it possible to correct them.

**Figure 8:**
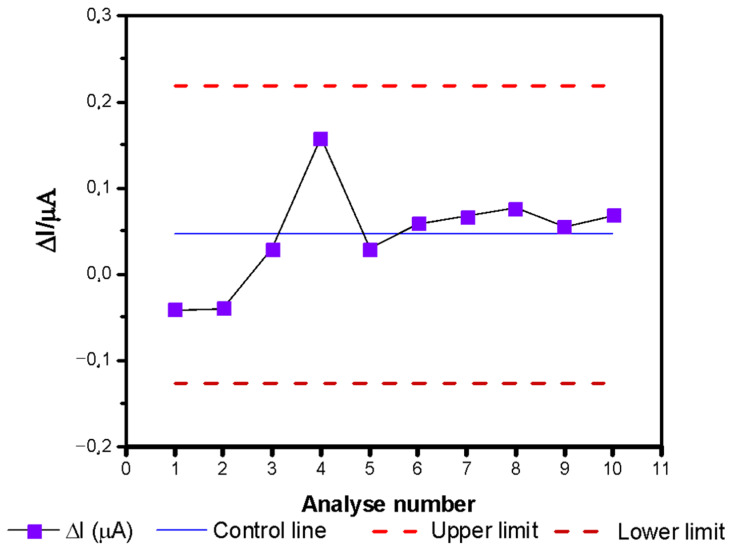
Statistical control chart for monitoring the biosensor stability.

Figure 8 shows the statistical control chart, built from chronoamperometric measurements, obtained with the same biosensor, in a medium containing 9.9 µmol·L^−1^ HX, in the absence of antioxidants, over the course of 7 months. The biosensor proved to be statistically stable in this period, with the control lines not being exceeded once.

## 4. Conclusions

The use of silk-screened sensors, whose working electrode contains graphite and the Prussian blue mediator (PB) proved to be efficient in determining the antioxidant capacity when the target molecule is hydrogen peroxide. This was possible due to the catalytic properties of this modifier, at the time of its reduction. The use of Meldola blue combined with Reinecke salt (MBRS) was not effective in the studied conditions. However, the possibility of future applications should not be ruled out, due to its high potential in electron transfer processes.

The method of immobilization by occlusion/entrapment of the enzyme xanthine oxidase (XOD) in polymeric PVA-AWE film on the surface of the carbon paste electrode modified with PB proved to be simple and efficient. However, the polymerization conditions must be properly controlled because a small variation in the polymerization time has considerably affected the analytical response of the biosensor

The elimination of the constant agitation process, during chronoamperometric measurements, for example, resulted in an increase in the linear range reached by the device. If higher levels of enzyme activity and substrate content are used, but always below the level of kinetic saturation, it is believed that the linear region can be further expanded.

The biosensor showed high stability and showed promise in determining the antioxidant capacity of teas and/or drugs that are sources of vitamin C and can also be used to detect fraud. Its use can be expanded to assess the antioxidant potential of fresh or processed foods and also to control different products used for their neuroprotective effects.

The developed biosensors could be used as possible tools to the monitoring of reactive oxygen species, the free radicals in different samples, as plants extracts, drugs, or other biological liquids, aiming to obtain a correlation between the index obtained from these indicators with the oxidative stress levels in the samples.

## Figures and Tables

**Table 1:** Shows the analytical efficiency of the biosensor in terms of R^2^, sensitivity, and linear range, under constant or controlled agitation.

Equation	R^2^	Sensitivity	Linear Range (µmol·L ^−1^)
I ^1^=−137.91+(−9.98) × [HX]	0.994	−9.98	0–50
I ^2^=−165.43+(−9.29) × [HX]	0.991	−9.29	0–69
I ^3^=−102.36+(−8.33) × [HX]	0.995	−8.33	0–69
I ^4^=−0.12+(−0.02) × [HX]	0.999	−0.02	20–136

^1^ Constant agitation (300 rpm); ^2^ 15 s agitation, 15 s rest, 75 s current measurement; ^3^ 30 s agitation, 15 s rest, 60 s current measurement; ^4^ 15 s agitation, 15 s rest, 60 s current measurement. A new chronoamperometric run was performed at each substrate concentration.

## Data Availability

Not applicable.

## Supplementary Materials

The following are available online at https://www.mdpi.com/2076-3921/10/2/324/s1, Figure S1: Distribution of the references concerning antioxidant capacity detection, according with publication years—since 1997; Figure S2: Distribution of the references concerning antioxidant capacity detection, according with document type—since 1997; Figure S3: Distribution of the references concerning antioxidant capacity detection, according with source titles—since 1997; Figure S4: Distribution of the references concerning antioxidant capacity detection, according research area—since 1997; Figure S5: H-index related to the ISI WOS references dealing with antioxidant capacity detection—since 1997.

## References

[1] Nunez-Selles A.J. (2005). Antioxidant therapy: Myth or reality?. J. Braz. Chem. Soc..

[2] Barreiros A., David J.M., David J.P. (2006). Oxidative stress: Relations between the formation of reactive species and the organism’s defense. Quim. Nova.

[3] Gliszczynska-Swiglo A. (2006). Antioxidant activity of water soluble vitamins in the TEAC (trolox equivalent antioxidant capacity) and the FRAP (ferric reducing antioxidant power) assays. Food Chem..

[4] Vasconcelos S.M.L., Goulart M.O.F., Moura J., Manfredini V., Benfato M.D.S., Kubota L.T. (2007). Reactive oxygen and nitrogen species, antioxidants and markers of oxidative damage in human blood: Main analytical methods for their determination. Quim. Nova.

[5] Halliwell B. (2012). Free radicals and antioxidants: Updating a personal view. Nutr. Rev..

[6] Carlsen M.H., Halvorsen B.L., Holte K., Bohn S.K., Dragland S., Sampson L., Willey C., Senoo H., Umezono Y., Sanada C. (2010). The total antioxidant content of more than 3100 foods, beverages, spices, herbs and supplements used worldwide. Nutr. J..

[7] Li S., Li S.K., Gan R.Y., Song F.L., Kuang L., Li H.B. (2013). Antioxidant capacities and total phenolic contents of infusions from 223 medicinal plants. Ind. Crop. Prod..

[8] Deng G.F., Lin X., Xu X.R., Gao L.L., Xie J.F., Li H.B. (2013). Antioxidant capacities and total phenolic contents of 56 vegetables. J. Funct. Foods.

[9] Li A.N., Li S., Li H.B., Xu D.P., Xu X.R., Chen F. (2014). Total phenolic contents and antioxidant capacities of 51 edible and wild flowers. J. Funct. Foods.

[10] Xu D.P., Li Y., Meng X., Zhou T., Zhou Y., Zheng J., Zhang J.J., Li H.B. (2017). Natural Antioxidants in Foods and Medicinal Plants: Extraction, Assessment and Resources. Int. J. Mol. Sci..

[11] Becker M.M., Nunes G.S., Ribeiro D.B., Silva F., Catanante G., Marty J.L. (2019). Determination of the Antioxidant Capacity of Red Fruits by Miniaturized Spectrophotometry Assays. J. Braz. Chem. Soc..

[12] Becker M.M., Ribeiro E.B., Marques P., Marty J.L., Nunes G.S., Catanante G. (2019). Development of a highly sensitive xanthine oxidase-based biosensor for the determination of antioxidant capacity in Amazonian fruit samples. Talanta.

[13] Pisoschi A.M., Negulescu G.P. (2011). Methods for total antioxidant activity determination: A review. Biochem. Anal. Biochem..

[14] Rodriguez-Amaya D.B. (2011). A Guide to Carotenoid Analysis in Foods.

[15] Lates V., Marty J.L., Popescu I.C. (2011). Determination of Antioxidant Capacity by Using Xanthine Oxidase Bioreactor Coupled with Flow-through H2O2 Amperometric Biosensor. Electroanalysis.

[16] Pereira A.C., de Santos A.S., Kubota L.T. (2002). Tendências em modificação de eletrodos amperométricos para aplicações eletroanalíticas. Quim. Nova.

[17] Hayat A., Marty J.L. (2014). Disposable Screen Printed Electrochemical Sensors: Tools for Environmental Monitoring. Sensors.

[18] Hoshi T., Saiki H., Anzai J. (2003). Amperometric uric acid sensors based on polyelectrolyte multilayer films. Talanta.

[19] Nunes G.S., Badea M., Medel M.L., Noguer T., Marty J.L. (2008). Ultrasensitive biosensors for the detection of insecticide residues in fruit juices. Bull. Transilv. Univ. Bras. Med. Sci. Ser..

[20] Saleem M., Yu H.J., Wang L., Zain ul A., Khalid H., Akram M., Abbasi N.M., Huang J. (2015). Review on synthesis of ferrocene-based redox polymers and derivatives and their application in glucose sensing. Anal. Chim. Acta.

[21] Ricci F., Palleschi G. (2005). Sensor and biosensor preparation, optimisation and applications of Prussian Blue modified electrodes. Biosens. Bioelectron..

[22] Rosatto S.S., Freire R.S., Durán N., Kubota L.T. (2001). Biossensores amperométricos para determinação de compostos fenólicos em amostras de interesse ambiental. Quim. Nova.

[23] Pandey P.C., Pandey A.K. (2013). Novel synthesis of Prussian blue nanoparticles and nanocomposite sol: Electro-analytical application in hydrogen peroxide sensing. Electrochim. Acta.

[24] Varvari L., Popescu I.C. (2010). New method for antioxidant activity evaluation using a H2O2 amperometric sensor. Rev. Roum. Chim..

[25] Stoytcheva M., Zlatev R., Navarro F.F.G., Velkova Z., Gochev V., Montero G., Bautistaa A.G.A., Toscano-Palomar L. (2016). PVA-AWP/tyrosinase functionalized screen-printed electrodes for dopamine determination. Anal. Methods.

[26] Banerjee S., Sarkar P., Turner A.P.F. (2013). Amperometric biosensor based on Prussian Blue nanoparticle-modified screen-printed electrode for estimation of glucose-6-phosphate. Anal. Biochem..

[27] Bhattacharyya A., Chattopadhyay R., Mitra S., Crowe S.E. (2014). Oxidative stress: An essential factor in the pathogenesis of gastrointestinal mucosal diseases. Physiol. Rev..

[28] Nakamura T., Silva F.S., Silva DX da Souza MW de Moya H.D. (2013). Determinação da atividade antioxidante e do teor total de polifenol em amostras de chá de ervas comercializadas em sachets. Abcs Health Sci.

[29] Chung V., Liu L., Bian Z., Zhao Z., Fong W.L., Kum W.F., Gao J., Li M. (2006). Efficacy and safety of herbal medicines for idiopathic Parkinson’s disease: A systematic review. Mov. Disord..

[30] More S.V., Kumar H., Kang S.M., Song S.Y., Lee K., Choi D.K. (2013). Advances in neuroprotective ingredients of medicinal herbs by using cellular and animal models of Parkinson’s disease. Evid.-Based Complement. Altern. Med..

[31] Raman S., Asle-Rousta M., Rahnema M. (2020). Protective effect of fennel, and its major component trans-anethole against social isolation induced behavioral deficits in rats. Physiol. Int..

[32] Han A.Y., Lee H.S., Seol G.H. (2016). Foeniculum vulgare Mill. increases cytosolic Ca2+ concentration and inhibits store-operated Ca2+ entry in vascular endothelial cells. Biomed. Pharm..

[33] Bhatti S., Ali Shah S.A., Ahmed T., Zahid S. (2018). Neuroprotective effects of Foeniculum vulgare seeds extract on lead-induced neurotoxicity in mice brain. Drug Chem. Toxicol..

[34] Mahboubi M. (2019). Foeniculum vulgare as Valuable Plant in Management of Women’s Health. J. Menopausal Med..

[35] Amsterdam J.D., Li Q.S., Xie S.X., Mao J.J. (2020). Putative Antidepressant Effect of Chamomile (Matricaria chamomilla L.) Oral Extract in Subjects with Comorbid Generalized Anxiety Disorder and Depression. J. Altern. Complement. Med..

[36] McKay D., Blumberg J. (2006). A Review of the bioactivity and potential health benefits of chamomile tea (*Matricaria recutita* L.). Phyther. Res..

[37] López V., Martín S., Gómez-Serranillos MPCarretero M., Jäger A., Calvo M. (2010). Neuroprotective and neurochemical properties of mint extracts. Phyther. Res..

[38] Hanafy D.M., Burrows G.E., Prenzler P.D., Hill R.A. (2020). Potential role of phenolic extracts of mentha in managing oxidative stress and Alzheimer’s disease. Antioxidants.

[39] De Bula M. Cimegripe^®^ 77 C. https://remediobarato.com/cimegripe-77c-bula-completa--cimed-industria-de-medicamentos-ltda--para-o-profissional.html#verpdf.

[40] Penteado M.D.V.C. (2021). Vitaminas: Aspectos Nutricionais, Bioquímicos, Clínicos e Analíticos.

[41] de Souza A.V., da Vieira M.R.S., Putti F.F. (2018). Correlações entre compostos fenólicos e atividade antioxidante em casca e polpa de variedades de uva de mesa. Braz. J. Food Technol..

[42] Guo C., Yang J., Wei J., Li Y., Xu J., Jiang Y. (2003). Antioxidant activities of peel, pulp and seed fractions of common fruits as determined by FRAP assay. Nutr. Res..

[43] Badea M., Chiperea S., Bălan M., Floroian L., Restani P., Marty J.L., Iovan C., Ţiţ D.M., Bungău S., Taus N. (2018). New approaches for electrochemical detection of ascorbic acid. Farmacia.

