# Supplementary material for: Determination of the Antioxidant Activity of Samples of Tea and Commercial Sources of Vitamin C, Using an Enzymatic Biosensor
Source: Antioxidants (Basel). 2021 Feb 22;10(2):324. doi: 10.3390/antiox10020324 (PMC7927098; doi:10.3390/antiox10020324)
Supplement: Supplementary file 1 [file antioxidants-10-00324-s001.pdf]

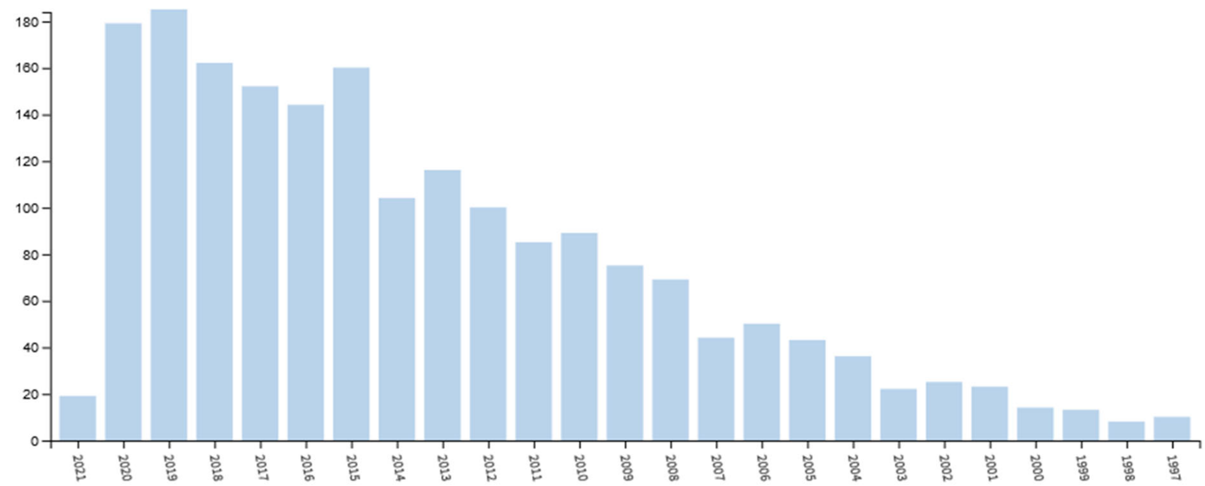

Figure 1. Distribution of the references concerning antioxidant capacity detection, according with publication years – since 1997

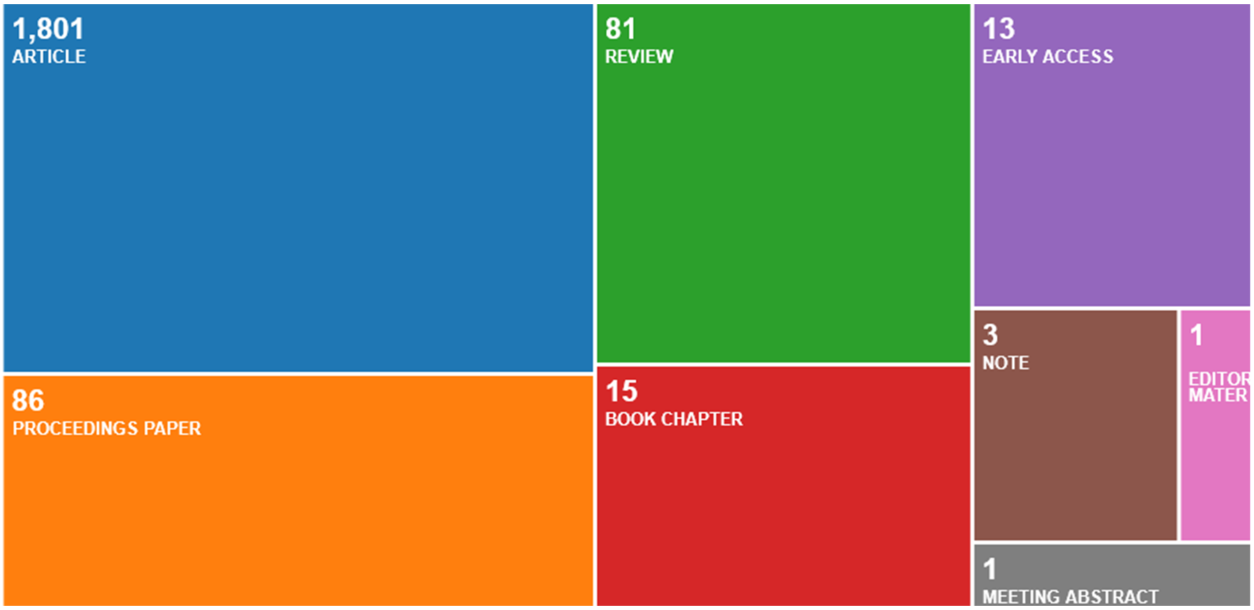

Figure 2. Distribution of the references concerning antioxidant capacity detection, according with document type – since 1997

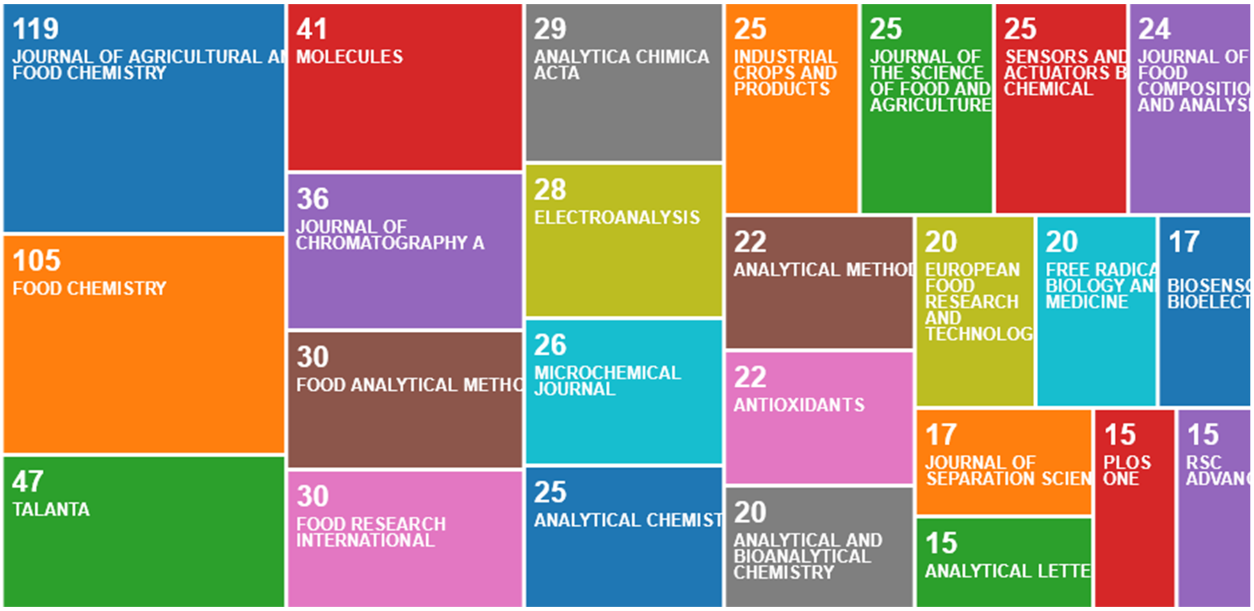

Figure 3. Distribution of the references concerning antioxidant capacity detection, according with source titles – since 1997

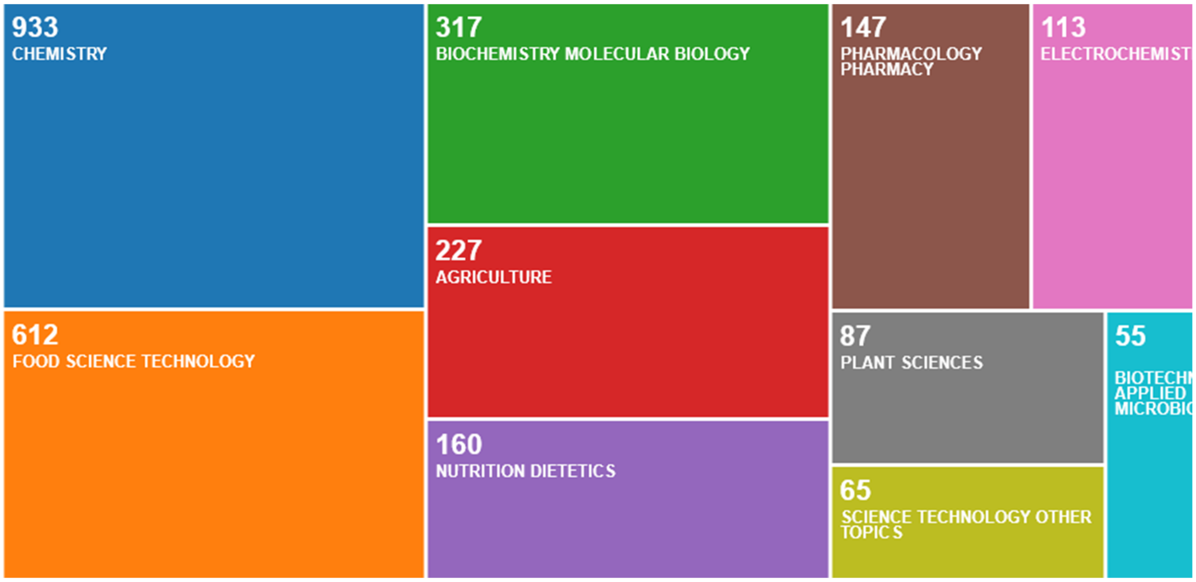

Figure 4. Distribution of the references concerning antioxidant capacity detection, according research area – since 1997

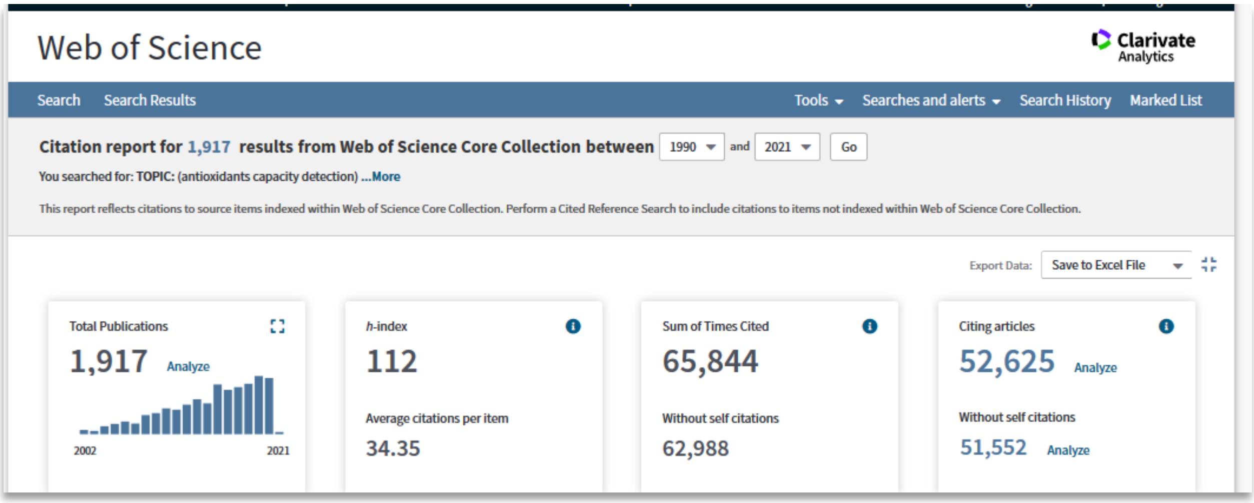

Figure 5. H-index related to the ISI WOS references dealing with antioxidant capacity detection – since 1997
